# Intraoperative Hemodynamic Instability in a Patient With Ebstein's Anomaly Complicated With Eisenmenger Syndrome

**DOI:** 10.1155/cric/8283566

**Published:** 2024-12-17

**Authors:** Leonardo A. Marquez Roa, Jorge Araujo-Duran, Richard Hofstra, Jibran Ikram, Sabry Ayad

**Affiliations:** Department of Anesthesiology and Pain Management, Cleveland Clinic, Cleveland, Ohio, USA

**Keywords:** Ebstein's anomaly, Eisenmenger syndrome, intraoperative hemodynamic instability, intraoperative hypoxemia, positive end-expiratory pressure (PEEP), right-to-left shunting, transesophageal echocardiography (TEE)

## Abstract

Ebstein's anomaly is a rare congenital displacement of the tricuspid valve resulting in atrialization of the right ventricle. About half of the patients with Ebstein's anomaly also have atrial septal defects, which may lead to chronic shunting and development of Eisenmenger syndrome. We describe a case of a sexagenarian male patient with a history of Ebstein's anomaly complicated with Eisenmenger syndrome undergoing robotic laparoscopic adrenalectomy who presented hemodynamic instability, hypoxemia, and likely right-to-left shunting intraoperatively, as well as the actions taken to correct it and have a successful outcome. Perioperative management of adult patients with congenital heart defects is complex and requires careful monitoring. When available, intraoperative transesophageal echocardiography is strongly recommended. To prevent right-to-left shunting, maintaining elevated systemic vascular resistance with the use of vasopressors and low positive end-expiratory pressure (PEEP) ventilation is critical.

## 1. Introduction

Ebstein's anomaly is a congenital disorder of the tricuspid valve in which the attachments of the septal and posterior leaflets of the tricuspid valve are apically displaced. It presents in approximately one in 200,000 live births, accounting for less than 1% of all cases of congenital heart disease [1, 2]. Over 50% of patients with Ebstein's anomaly also present with atrial septal defects (ASDs) such as patent foramen ovale (PFO) which might cause shunting [3]. Chronic left-to-right shunting may produce pulmonary vascular remodeling secondary to increased pulmonary blood flow, which over time leads to pulmonary hypertension. This increase in the mean pulmonary arterial pressure may reverse the shunt (right-to-left) leading to hypoxemia and cyanosis, which is known as Eisenmenger syndrome [4]. Patients with Eisenmenger syndrome having noncardiac surgery may have up to 30% increased mortality risk [5]. Perioperative management has improved in the last 20 years. Nonetheless, it is still considered challenging, and the choice of anesthetic management should be tailored to the hemodynamic needs of each patient [6].

## 2. Case Description

Our patient is a sexagenarian male with a history of Ebstein's anomaly and an ASD complicated with Eisenmenger syndrome. The patient was scheduled for robotic laparoscopic left adrenalectomy due to an enlarged nodule, suspicious for metastatic renal cell carcinoma (clear cell type), and treated with partial nephrectomy 2 years before the current episode. Additional relevant history included mild pulmonary artery (PA) hypertension, coronary artery disease, systemic hypertension, and prostate cancer with metastatic disease to the bone, currently in remission. Prior to surgery, the patient reported no significant shortness of breath, chest pain, palpitations, syncope, or lower extremity edema. His vital signs were blood pressure (BP) of 126/78 mmHg, heart rate (HR) of 110 bpm, and oxygen saturation (SpO_2_) of 91% on room air. Cardiac examination revealed regular rhythm, point of maximal impulse not displaced, split second heart sound (S2), positive fourth heart sound (S4), and a 1/6 to 2/6 systolic murmur. Cyanosis, jugular venous distension, peripheral edema, and organomegaly were not evidenced. Electrocardiogram showed right bundle branch block and diffuse ST and T wave changes. On the day of surgery, the patient presented hemodynamically stable. He was premedicated with midazolam (2 mg) intravenous (IV). Right radial artery catheterization was performed for hemodynamic monitoring. He received propofol (150 mg) and etomidate (10 mg) IV for induction together with phenylephrine (100 mcg) IV for attenuation of postinduction hypotension, rocuronium for muscle paralysis, and isoflurane for maintenance of anesthesia.

**Figure 1:**
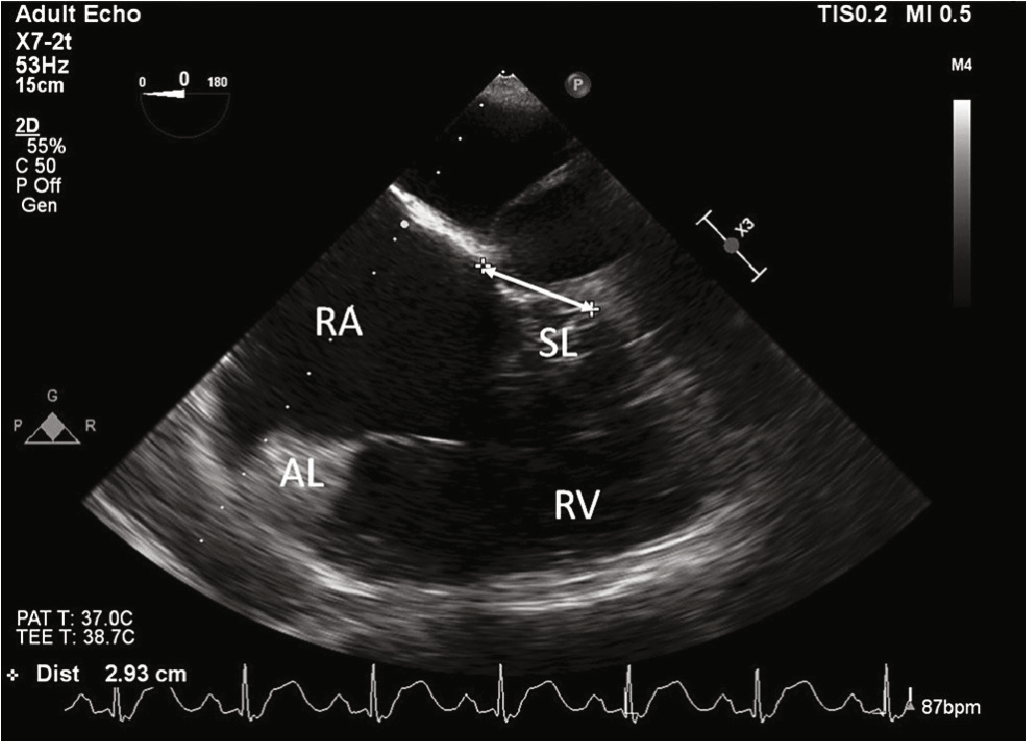
Midesophageal four-chamber view of TEE evidencing an apical displacement of 2.93 cm of the septal leaflet of the tricuspid valve (double-headed arrow). RA: right atrium; RV: right ventricle; SL: septal leaflet of the tricuspid valve; AL: anterior leaflet of the tricuspid valve.

**Figure 2:**
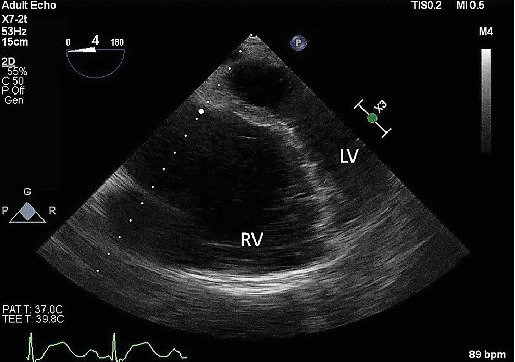
Midesophageal four-chamber view of TEE showing severe dilation of the right ventricle compared to the left ventricle. RV: right ventricle; LV: left ventricle.

**Figure 3:**
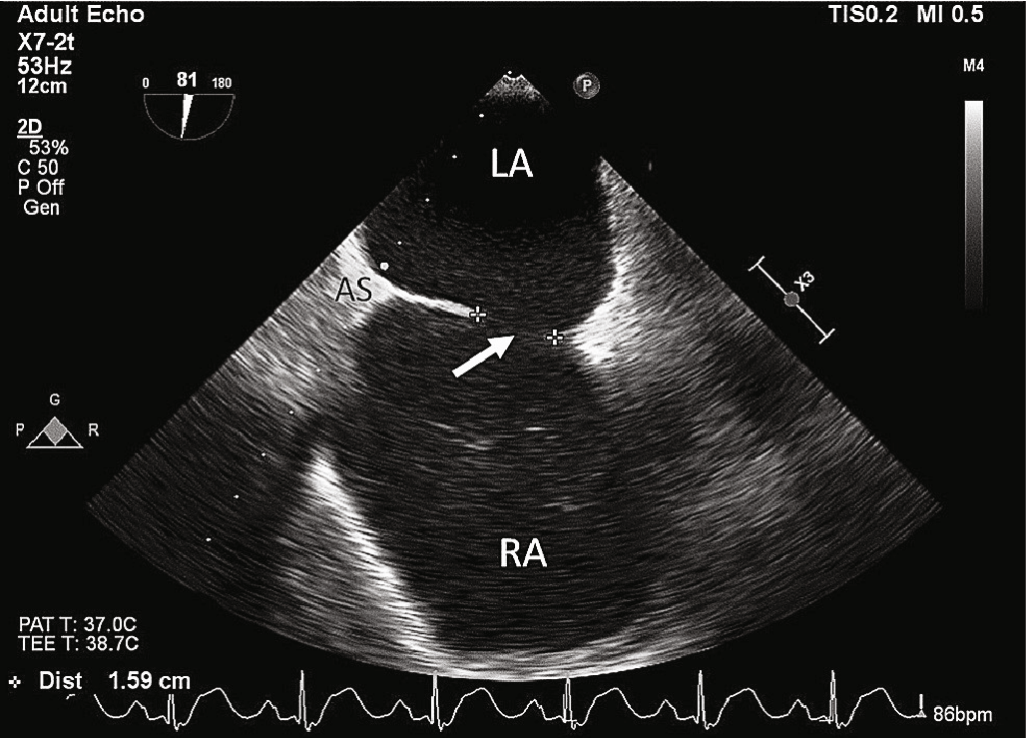
Midesophageal bicaval view of TEE showing atrial septal defect of 1.59 cm (arrow). RA: right atrium; LA: left atrium; IAS: interatrial septum.

An intraoperative assessment was conducted using transesophageal echocardiogram (TEE), which revealed a septal leaflet that was apically displaced by 2.9 cm (see Figure 1), consistent with Ebstein's anomaly. The examination also revealed moderate tricuspid regurgitation and severe dilation of the right atrium and ventricle (see Figure 2), with mildly decreased right ventricular systolic function. The left ventricle was found to be small but with normal function (see Figure 2). Trace aortic regurgitation was noted, and an ASD measuring 1.59 cm was identified (see Figure 3), which exhibited bidirectional shunting. The patient was positioned in the right lateral decubitus position, and access to the abdomen was obtained for carbon dioxide (CO_2_) insufflation. At the time of incision, vital signs showed BP of 135/80 mmHg, HR of 90 bpm, and SpO_2_ of 100% with a fraction of inspired oxygen (FiO_2_) of 92% and remained stable throughout insufflation. Ventilation settings were set to a positive end-expiratory pressure (PEEP) of 5 cmH_2_O and a tidal volume of 500 mL. Subsequently, the patient received additional 50 mg of propofol and 20 mg of rocuronium. However, a further decrease in SpO_2_ was observed immediately, and the anesthesia team decided to increase the PEEP to 8 cmH_2_O and then 11 cmH_2_O to improve oxygenation. Unfortunately, this maneuver resulted in a dramatic drop in SpO_2_ to 79%, and a reversal of the atrial shunt was suspected. To manage the ongoing hypoxemia due to the suspected right-to-left shunt via ASD, the PEEP was decreased to 4 cmH_2_O, intra-abdominal CO_2_ insufflation was discontinued, and vasopressors were administered, as the MAP (mean arterial pressure) had decreased to 90 mmHg. The goal was to increase systemic vascular resistance (SVR), and lower pulmonary arterial pressure. After these measures, hemodynamic stability was achieved. CO_2_ insufflation to the abdomen was performed again at a pressure of 8 mmHg, and the SpO_2_ was stabilized at > 94% for the rest of the procedure, with a MAP above 100 mmHg. Neuromuscular blockade was reversed with sugammadex 250 mg, and the patient was taken to the surgical ICU for observation in a stable condition.

## 3. Discussion

Some undiagnosed congenital malformations may remain asymptomatic until adulthood and might be considered benign. However, when there is chronic and progressive mismatch between systemic and pulmonary circulation, clinical manifestations might become evident and substantial [6]. The patient was diagnosed with Ebstein's anomaly 2 years before the current episode, during a preoperative cardiac clearance prior to laparoscopic surgery for suspected renal cancer. At that time, he presented with severe postoperative hypoxemia. Studies evidenced elevated pulmonary arterial pressure and right-to-left shunting, leading to the diagnosis of Eisenmenger syndrome. Although surgical correction of the ASD and tricuspid valve repair were considered, cardiac surgery was postponed due to the urgency of his cancer treatment. The anesthetic management of a patient with Ebstein's anomaly complicated with Eisenmenger syndrome is challenging and requires a deep understanding of the physiopathology involved. It is more important to focus on the hemodynamic stability of the patient rather than on specific techniques or drugs [7]. The main goal is the maintenance or elevation of the SVR and prevention of a pulmonary hypertensive crisis to avoid increasing the right-to-left shunt during the perioperative period [5, 7]. The use of vasopressors at the time of induction is strongly recommended, since significant hypotension may occur [5]. Intraoperative TEE has been shown to be a useful tool for assessing cardiac anatomy and function in patients during surgery [3, 5]. In our patient, TEE allowed us to visualize the bidirectional flow of the shunt and assess the degree of tricuspid regurgitation, providing valuable information for clinical decision-making. However, due to space limitations and the lateral decubitus position required for the surgery, it was not feasible to use TEE throughout the entire procedure. In patients with intracardiac shunting, an increase in the inspired oxygen concentration has minimal effect in improving oxygenation [6]. Acute hemodynamic decompensation may be signaled by oxygen desaturation and worsening or refractory hypotension [5]. A relative decrease in MAP was observed in our patient as the SpO_2_ was declining rapidly. Hence, a primary goal of anesthetic management in patients with pulmonary hypertension secondary to Eisenmenger syndrome is to minimize increases in pulmonary vascular resistance (PVR) and to maintain SVR [8]. High PEEP increases PVR [7]. In patients with Eisenmenger syndrome, increasing the PEEP could be a trigger for right-to-left shunting and hypoxemia, since the consequent increase in PVR would translate into elevated afterload in the right heart, contributing to a change in the direction of the flow (right-to-left) and acute decompensation [9]. A previous case report of a patient having an intraoperative right-to-left shunt reported a complete resolution of the shunt with normal SpO_2_ after a reduction of the PEEP to 3 cm H_2_O [10]. A low PEEP (4 cm H_2_O) was well tolerated in this patient once the SVR was increased and improved the patient's oxygenation during the surgery. The use of vasopressors is one of the cornerstones in maintaining an adequate SpO_2_ in patients with intracardiac shunt, as the increase in SVR may prevent right-to-left shunting. In our case, this was clearly evidenced by the normalization of the SpO_2_ (> 94%) when phenylephrine was used to increase the MAP after the acute decompensation. However, because low-dose vasopressin increases SVR without significantly increasing PVR, it might be preferable in certain patients to use vasopressin rather than phenylephrine to maintain the SVR [11]. Increased intra-abdominal pressure (IAP) may also contribute to the hemodynamic changes seen in this patient. Elevated IAP increases peak inspiratory pressure and reduces respiratory compliance due to restricted movement of the diaphragm [12]. For this reason, CO_2_ insufflation was paused with the goal of improving respiratory compliance and reducing the elevated pulmonary pressure, decreasing the factors that were contributing to the right-to-left shunting in this patient. Additionally, it is suggested to avoid prone and lateral decubitus positions since they affect the preload and afterload and impede ventilation, having a deleterious effect on right-to-left shunting [13]. Given that TEE monitoring was not possible throughout the entire procedure, one alternative for future cases is the use of a PA catheter for tight perioperative hemodynamic monitoring. The American Society of Anesthesiologists recommends PA catheter monitoring for selected surgical patients to reduce perioperative complications by providing immediate access to critical hemodynamic data [14]. The decision to use PA catheter monitoring should be made based on the risk-benefit analysis for every individual patient.

## 4. Conclusion

Adult patients with congenital heart defects require careful management during the perioperative period due to their complex pathophysiology, which can lead to hypoxemia and hemodynamic instability. When available, intraoperative TEE is a useful tool to monitor hemodynamic status and detect any complications promptly. Maintaining an elevated SVR and a low PVR is essential to prevent right-to-left shunting. Therefore, the use of vasopressors is recommended along with low PEEP ventilation settings. Decreasing IAP should also be considered when applicable as it can reduce pulmonary pressure and avoid right-to-left shunting.

## Data Availability

The data that support the findings of this study are available on request from the corresponding author. The data are not publicly available due to privacy or ethical restrictions.
